# A spatio-temporal model of embolism propagation in leaf vein networks

**DOI:** 10.1093/aobpla/plaf020

**Published:** 2025-04-12

**Authors:** Diane Lu, Chris M Smith-Martin, Robert Muscarella, María Uriarte, Tian Zheng

**Affiliations:** Department of Statistics, Columbia University, 1255 Amsterdam Ave, New York, NY 10027, United States; Department of Plant and Microbial Biology, University of Minnesota, 140 Gortner Laboratory 1479 Gortner Avenue, St. Paul, MN 55108, United States; Plant Ecology and Genetics, Institute for Ecology and Genetics, Uppsala University, Norbyvägen 18 D, Uppsala 75236, Sweden; Department of Ecology, Evolution and Evolutionary Biology, Columbia University, 1014 Schermerhorn Extension, New York, NY 10027, United States; Department of Statistics, Columbia University, 1255 Amsterdam Ave, New York, NY 10027, United States

**Keywords:** embolism, leaf hydraulics, spatial survival analysis, tropical tree drought resistance, xylem vulnerability curves

## Abstract

Leaf veins hydrate and sustain leaf tissue for photosynthesis. During drought and freeze events, embolisms can form in xylem conduits, ceasing the transport of water. Understanding the formation and propagation of embolisms is crucial to predicting species’ responses to a changing climate. We develop a novel spatio-temporal model for embolism propagation, explore the dynamics of xylem cavitation through spatial survival analysis modelling, and quantitatively examine the relationship between leaf venation features and embolism propagation. Our work models embolism propagation through spatial survival modelling, allowing us to compare the importance of different factors (vein thickness and spatial dependency) in embolism formation and predict future embolism occurrences. The model is fitted to published spatio-temporal embolism data for leaves of eight evergreen tropical tree species collected using the optical vulnerability technique. Results derived from our analyses shed light on the role of venation patterns on embolism formation. We found that incorporating spatial dependency reduces uncertainty in estimating vulnerability curves and posterior predictive error, thus supporting the notion that embolism formation exhibits spatial dependence. Specifically, the likelihood of embolism in a vein segment increases when adjacent veins are affected. Furthermore, including vein thickness information improves the prediction of future embolism events. Additionally, our model revealed that leaves with more connected vein networks (i.e. the degree of connectivity) exhibit a more pronounced pattern of embolizing from thicker to thinner veins. Understanding the formation and propagation of embolisms is crucial to understanding species’ responses to a changing climate. The proposed model provides a statistical tool to extract quantifiable insights on embolism propagation and how it is associated with observable leaf features, such as network connectivity. This approach allows for a systematic assessment of species’ responses to a drying climate.

## Teaser

Our paper explores the complex spatio-temporal dynamics of embolism formation in leaf vein networks and its relationship to leaf venation features. Applying a novel spatial survival model to data from evergreen trees, we show that embolism formation is spatially dependent, with embolisms more likely in adjacent veins. Incorporating vein thickness improves predictions, revealing that thicker veins embolize first. Notably, leaves with more connected vein networks exhibit a stronger pattern of embolizing from thicker to thinner veins. These findings highlight the role of venation structure in embolism progression and provide a powerful tool for assessing species' resilience to drought and freeze events.

## Introduction

Leaf veins supply water to leaf tissue to maintain hydration and sustain photosynthesis (Sack and Holbrook 2006). Water is transported to the leaves in a continuous column driven by a tension gradient generated when water evaporates from the open leaf stomata, which increases the tension on the column, generating the flow of water from the roots to the leaves (Dixon and Joly 1895; Dixon 1914; Scholander 1972; Zimmermann 1983; Boyer 1985). During drought, tension in the water column increases, causing gas bubbles to expand and embolize xylem conduits, which can then no longer transport water (Tyree and Sperry 1989). The xylem in leaves is thought to be at the greatest risk of embolism formation and propagation because water in the leaves is under the greatest tension of the whole plant (Tyree and Ewers 1991). Leaf architecture generally reflects a trade-off between maximizing the efficiency of water movement and safety to limiting embolism formation and propagation, with larger leaf veins that transport water more efficiently also being more susceptible to embolisms (Hacke *et al.* 2006; Brodribb *et al.* 2016a).

Land plants exhibit highly variable leaf venation network architectures, from a singular vascular strand in some conifers, open net venation in ferns, predominantly parallel vines in monocots, to reticulated patterns in dicots (Blonder et al. 2020). Leaf veins are composed of bundles of xylem conduits, and the size, number, density, and network architecture of these conduits vary among groups of plants and can vary within a single leaf. Angiosperms have hierarchical, reticulate network venation architectures typically with large leaf xylem conduits arranged in bundles in the primary veins and highly reticulated small single conduits in tertiary veins (Sack and Scoffoni 2013; Scoffoni et al. 2017). Previous studies have found that embolism events start in the larger leaf veins in some species of ferns and in angiosperms such as oaks, eucalyptus, tomatoes, and wheat (Brodribb *et al.* 2016a, b; Skelton *et al.* 2017; Johnson *et al.* 2018; Skelton *et al.* 2021), supporting the hypothesis of a trade-off between hydraulic efficiency and safety (Brodribb *et al.* 2016a). These findings suggest that the complexity of vein network architecture plays a role in the degree of disruption to the water supply. For example, in plants with simpler vein network architecture, such as ferns or monocotyledons like palms, a single embolism event can propagate unrestricted to a larger proportion of a leaf’s venation network causing greater disruption to the leaf water supply (Brodribb *et al.* 2016a). However, in species with more complex vein network architecture such as those found in many dicotyledons, the propagation of embolisms is more restricted to a smaller proportion of a leaf’s vein network causing less disruption to the leaf water supply.

While previous studies have established a link between vein size and embolism formation (Brodribb *et al.* 2016a, b; Skelton *et al.* 2017; Johnson *et al.* 2018; Skelton *et al.* 2021; Isasa *et al.* 2023) and hypothesized spatial proximity of vessel conduits plays a role in embolism formation (Tyree and Sperry 1989), our research extends this investigation to a wider range of leaf venation features. This broader focus was driven by the hypothesis that additional vein characteristics, beyond just vein size, may significantly impact embolism formation (Sack and Scoffoni 2013).

To examine the relationship between leaf venation features (e.g. average vein density, the number of areoles, and vein connectivity) in addition to vein size and the spatio-temporal pattern of embolism propagation across a large dataset, two key challenges must be addressed. First, we need to automate the process of obtaining the leaf venation features, as manually analysing a large dataset would be excessively time-consuming and labour-intensive. Secondly, we require a model that can disentangle the potential driving factors (vein thickness and spatial dependency) of embolism propagation.

Although many studies have observed that higher vein orders tend to embolize earlier, these analyses relied on manual annotation of vein order, a method not scalable to large datasets (Brodribb *et al.* 2016a, b; Skelton *et al.* 2017; Johnson *et al.* 2018; Skelton *et al.* 2021). The work by Isasa et al. 2023 addressed this limitation by using vessel diameter obtained through automated leaf venation network segmentation software developed by Xu et al. 2021, and exploring the relationship it has with *P*_**50,**_ using linear mixed-effects model. Although this method allowed them to investigate the temporal aspect of vein size on embolism propagation, their focus remained on vein size, without exploring other aspects of venation features or the spatial aspect of embolism propagation.

Based on previous findings, our proposed survival model framework for modelling embolism propagation integrates three components: a basic temporal component, a spatial component, and a vein thickness component. This separation allows us to explicitly examine the relationships between these components and leaf venation features (e.g. average vein density, the number of areoles, and vein connectivity). By identifying associations between leaf venation features and embolism propagation factors, we can better predict embolism behaviour based on species’ venation network features. Moreover, with the separation of potential embolism factors (vein thickness and spatial dependency), our model enables us to investigate whether these factors influence the estimation of xylem vulnerability curves and the prediction of future embolism events, and how these factors differ across species. This approach also allows us to assess the relative importance of vein thickness and spatial dependency in embolism progression for each species.

Our proposed spatial survival model is designed to quantify the spatio-temporal patterns of leaf embolism propagation and to examine their relationships with leaf venation features. We developed three variations of these models, differing in their assumptions about the structure of spatial proximity and the inclusion of the vein thickness component. Here, we fit our models to eight evergreen wet tropical forest angiosperm tree species and address the following questions:

1) Do potential embolism propagation factors, such as vein thickness and spatial dependency, influence the estimation of xylem vulnerability curves and prediction of future embolism occurrences? Here, spatial dependency refers to the assumption that embolism events in neighbouring veins occur at similar times.
2) What relationships exist between the spatio-temporal dynamics of embolism propagation and leaf venation features (e.g. average vein density, the number of areoles, and vein connectivity)? Beyond the tendency of thicker veins to embolize earlier based on earlier empirical findings (Brodribb *et al.* 2016a, b; Skelton *et al.* 2017; Johnson *et al.* 2018; Skelton *et al.* 2021; Isasa *et al.* 2023 ), it remains unclear what is the connection between the leaf vein network connectivity and embolism occurrences.
3) As a robust model should not only discover new insights but also validate existing findings, we aim to see whether our model confirms that thicker veins tend to embolize earlier and how the temporal aspect of our model compares to P_**50**_ of previously published leaf optical vulnerability curves (Smith-Martin et al. 2022, 2023). This allows us to establish the reliability of our model in both supporting known phenomena and exploring new aspects of embolism propagation.

Understanding embolism formation plays a pivotal role in deciphering how plants respond to drought. While previous studies have successfully summarized temporal information of embolism formation through vulnerability curves and investigated the connection between these curves and vein thickness, they encountered limitations in conventional statistical methodologies (e.g. Pearson correlation coefficient, linear mixed-effects model). These limitations precluded the consideration of the spatial dependence aspect of embolism formation and hindered their capacity to model different driving factors of the embolism formation process. Our proposed spatial survival model surmounts these challenges. It presents an approach to modelling embolism formation that accounts for both the spatial dependence among veins and the thickness of these veins simultaneously, enriching our understanding of the spatio-temporal dynamics governing embolism formation.

## Materials and methods

### Replication statement

**Table:** 

Scale of inference	Scale at which the factor of interest is applied	Number of replicates at the appropriate scale
Individual	Individual	4–8 individuals per species
Species	Species	8 species

### Optical vulnerability curves

We used previously published leaf optical vulnerability curves (Smith-Martin et al. 2022, 2023) that were collected at Luquillo Experimental Forest in El Yunque National Forest (EYNF) located in northeastern Puerto Rico. The EYNF is classified as subtropical wet forest in the Holdridge life zone system (Ewel and Whitmore 1973), with mean annual precipitation ca. 3500 mm yr^-1^ and rainfall typically exceeding 100 mm in all months, making this forest largely aseasonal. For eight common evergreen tree species in the EYNF, xylem vulnerability to embolism on four to eight individuals per species (Table 1) was measured using the optical vulnerability technique (Brodribb *et al.* 2016a). See details in (Smith-Martin et al. 2022, 2023).

**Table 1.** List of species used in this study, code used in figures, their families, and number of individual trees measured per species.

Species	Code	Family	Num. individuals
*Alchornea latifolia*	ALCLAT	Euphorbiaceae	4
*Casearia arborea*	CASARB	Flacourtiaceae	8
*Cecropia schreberiana*	CECSCH	Moraceae	7
*Drypetes glauca*	DRYGLA	Euphorbiaceae	7
*Inga laurina*	INGLAU	Fabaceae	6
*Ocotea leucoxylon*	OCOLEU	Lauraceae	5
*Sloanea berteroana*	SLOBER	Elaeocarpaceae	8
*Tabebuia heterophylla*	TABHET	Bignonaceae	8

### Optical vulnerability image data

The optical vulnerability image data originates from the same source as the previously mentioned optical vulnerability curves (Smith-Martin et al. 2022, 2023). This data was originally obtained using the optical vulnerability technique (Brodribb *et al.* 2016a). The images have a resolution of 960 × 1280 pixels.

### Data processing

To make the optical vulnerability image data suitable for our proposed spatial survival model, several processing steps are essential. These data processing steps encompass the extraction of vein segments as the unit of analysis, the computation of vein thickness for each vein segment, the construction of a venation network to encode spatial dependencies between vein segments, and the determination of whether an embolism event occurred for each vein segment.

#### Vein segmentation process

**Figure 1.**
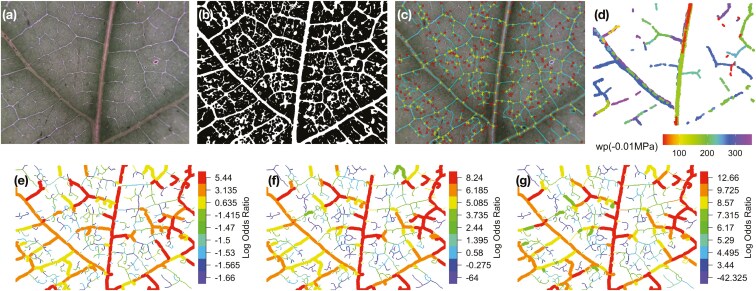
(a) Unprocessed image of a *Ocotea leucoxylon* sample. (b) Binarized image derived from the first image using phenoVein. (c) Locations of the branching points and ending points are highlighted by markers through phenoVein. (d) Visualization of embolism events during desiccation (i.e. increases in water potential, wp). Panels e–g show posterior frailties (measured by log odds ratio) for the three models considered, red colouring represents higher frailty. Higher frailties indicates a greater probability of embolism. Censored vein segments are visualized by thinner veins, while vein segments that embolized during observation are represented by thicker veins. (e) Spatial-Independent model. (f) Spatial-Dependent model. (g) Spatial-Dependent-Vein-Thickness model.

For the spatial model proposed in this paper, we treated "vein segments" as units of observation rather than pixels. This approach allowed us to study embolism propagation in a more biologically relevant manner. Furthermore, it reduced the sample size from roughly 1 000 000 pixels down to hundreds of vein segments per leaf sample, which substantially lowered the computational complexity of the analysis. We first identified the veins using phenoVein (Bühler et al. 2015), a semi-automated leaf vein segmentation software. The resulting image marks the positions of branching and ending points for each vessel in the sample. Using these markers, we defined a vein segment as the section of the vein occurring between two branching points, or between one branching point and one ending point (Fig. 1; Supplementary Fig. S1). We conducted a visual check after the automated segmentation to ensure accuracy. When we identified major issues with the segmentation quality (e.g. not correctly identifying the higher-order veins), manual corrections were made to improve the results. It is possible that one xylem conduit could be separated into multiple vein segments, or that one vein segment contains multiple xylem conduits (Mrad et al. 2021; Bouda et al. 2022). Nevertheless, this is the best approximation achievable given the present resolution of available images.

#### Vein thickness measurements

**Figure 2.**
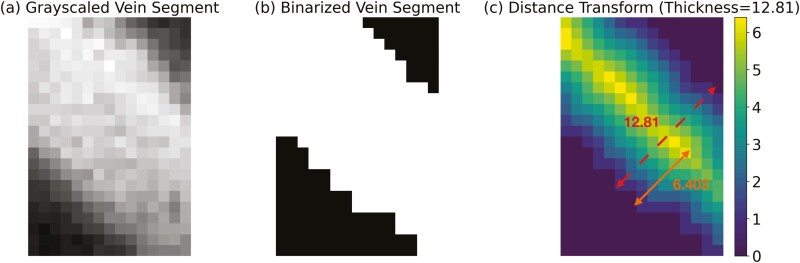
(a) A greyscale image example of a vein segment. (b) The binarized image of the vein segment. (c) The distance transform of the vein segment visualizes how far each vein pixel is from the background pixel. The pixels with higher values form the medial axis. The maximum Euclidean distance of the pixels on the medial axis to the nearest background pixel is 6.403, and hence the thickness for this vein segment is estimated as 2×6.403.

To explore the importance of vein thickness in embolism formation, we estimated the thickness of each vein segment using the binarized image generated through phenoVein during the vein segmentation process. An intuitive approach for estimating the segment’s thickness is to first find the medial axis of the vein segment and then calculate the width of the vein segment, which should be perpendicular to the medial axis. We identified the medial axis through the distance transform (van der Walt et al. 2014). The distance transformation displays the distance of a vein pixel to the closest background pixel for each vein pixel, and the vein pixels with higher distance transform values form the medial axis. Assuming the symmetric geometry of veins, vein thickness is twice the maximum pixel-level distance transform value of the medial axis (Fig. 2).

#### Venation network construction

To address whether spatial connectivity of the venation network plays a role in the spatio-temporal spread of embolisms, we defined neighbours as any two vein segments that were connected in the resulting image from phenoVein (Fig. 1).

#### Embolism events

In our study, if there was at least one embolized pixel in a particular vein segment for the image, we defined it as one embolism candidate event for that vein segment. After iteratively removing the spillover candidates detailed in Supplementary Notes S1, we mapped the embolized pixels to an embolism event. Our primary focus is the timing and location of these embolism events. To address the "where" aspect, we utilized spatial neighbouring information from the vein network. For the "when" dimension, we employed water potential associated with each embolism event, using its decrease over time as an implicit indicator of the event duration. Using the spatial neighbouring information and water potential of embolism events, we turned to our spatial survival model to unravel the spatio-temporal dynamics of these spatially referenced time-to-event embolism data.

### Spatial survival analysis

Survival analysis has long been a cornerstone in medical research for examining time-to-event data, such as the time-lapse until a critical event (e.g. death) occurs. In our study, we applied this methodology to time-to-event data related to embolism occurrence. An embolized vessel conduit remains permanently obstructed after an embolism occurrence, which makes survival analysis an appropriate method for our study. The survival probability of a vein segment at a given time is defined as the probability that the segment has not experienced an embolism event up to that specific time. We further establish a connection between survival functions and the commonly utilized vulnerability curves (Supplementary Notes S2). The connection reassures us that the spatial survival model we proposed is built upon the foundation of xylem analysis that others have laid.

Another reason why we chose to use survival analysis models for modelling the spatio-temporal dynamics of embolism formation is that survival models took censored vein segments into consideration. Right-censoring occurs when the event happens after the observation period ends. If we compare the veins extracted and observed embolism events (Fig. 1c, d), there are some vein segments that did not embolize during the observation period (i.e. right-censoring). On the other hand, left-censoring happens when the event takes place before the observation period starts. Although we do not have data to characterize left-censoring, the researchers who collected the data took great care to minimize water loss before measurements (Smith-Martin et al. 2022, 2023). Hence, we focussed on dealing with right-censoring in this study. If we ignore the censored vein segments, not only will the spatial dependence between vein segments be incorrectly captured, but also the estimation of the embolism formation probability would be overestimated (Watt et al. 1996; Leung et al. 1997). Specifically, if a censored vein segment is connected to many other veins, then failing to consider the censored data might cause spatial discontinuity in the estimated embolism formation probability. Therefore, we included both vein segments with observed events and right-censored vein segments in calculating our likelihood function for all of our models.

To perform spatial survival analyses, we first extracted time-to-event areal data from the raw leaf images obtained through the optical vulnerability technique (Brodribb *et al.* 2016b). The embolism event data for the spatial survival model can be summarized using an adjacency matrix for the venation network ($E=\{e_{ij}\})$, event indicator ($\delta =\{\delta_{i}:i=1,\;\ldots ,\;m\})$, and event water potential ($\tilde{t}=\{t_{i}\;i=1,\;\ldots ,\;m$}). Considering a total of *m* vein segments, $e_{ij}$ is 1 if the ith and the jth vein segments are neighbours; else $e_{ij}$ has a value of 0. $\delta_{i}$ is ith vein segment’s embolism event indicator, with a value of 1 if the ith vein segment embolized during the observation period and of 0 otherwise. $t_{i}$ is the water potential associated with the ith vein segment when $\delta_{i}=1$. If there is no embolism event observed in the ith vein segment (i.e. $\delta_{i}=0$), $t_{i}$ would be the water potential associated with the end of the observation period.

We proposed a spatial survival model framework that divides embolism formation into three fundamental components: temporal, spatial, and vein thickness. Based on this framework, we developed three proportional odds models (Zhou et al. 2020): the Spatial-Independent model, the Spatial-Dependent model, and the Spatial-Dependent-Vein-Thickness model. These models differ in their treatment of spatial and vein thickness components (Table 2). The Spatial-Dependent and Spatial-Dependent-Vein-Thickness models assume that neighbouring vein segments are likely to embolize around similar times, while the Spatial-Independent model does not have this model assumption. The Spatial-Dependent-Vein-Thickness model additionally incorporates the vein thickness component, unlike the Spatial-Independent and Spatial-Dependent models. By exploring different combinations of these components across the three models, we aim to gain a deeper understanding of how spatial proximity and vein thickness influence embolism propagation. The survival function for a vein segment at a given time t represents the probability that the segment has not experienced embolism up to time t. For the ith vein segment in the Spatial-Independent and Spatial-Dependent models, the survival function is defined as follows:

**Table 2.** Models used in this study, with the types of information each model leveraged. Temporal, spatial, and vein thickness information are encoded in the sequences of images that capture the process of embolism. All the models utilized temporal information. However, the KM estimator did not leverage spatial and vein thickness information. Furthermore, the Spatial-Dependent-Vein-Thickness model is the only model that explicitly made use of the vein thickness information.

Model name	Temporal	Spatial	Vein thickness
KM estimator (baseline)	Yes	No	No
Spatial-Independent model	Yes	Yes (IID prior, Eqn. 2)	No (Eqn. 1)
Spatial-Dependent model	Yes	Yes (ICAR prior, Eqn. 3)	No (Eqn. 1)
Spatial-Dependent-Vein-Thickness model	Yes	Yes (ICAR prior, Eqn. 3)	Yes (Eqn. 4)


$$\begin{array}{l} S_{i}\;\left(t\right)=\frac{e^{-v_{i}}\;S_{0,\theta }\left(t\right)}{1+\left(e^{-v_{i}}-1\right)S_{0,\theta }\left(t\right)} \end{array}$$ (1)

where $v_{i}$ is the ith vein segment’s unobserved spatial frailty. If the spatial frailty is large, the embolism events tend to occur earlier. $S_{0,\theta }\left(t\right)$ is the basic temporal component encoding the survival corresponding to no spatial dependence (i.e. $v_{i}=0)$. The term "frailty" refers to the random effect within a survival model, consistent with the terminology used in the survival analysis literature (Hougaard 1995).

However, the priors are different for the spatial frailty for the Spatial-Independent and Spatial-Dependent models. The priors were chosen to determine if (i) location is irrelevant to the probability of embolism formation, or (ii) the probability depends on embolism formation in neighbouring veins. The two different priors on spatial frailty are the "independent and identically distributed (IID) Gaussian prior" for the Spatial-Independent model and the "intrinsic conditionally autoregressive (ICAR) prior" for the Spatial-Dependent model.

IID prior:


$$\begin{array}{l} {\left(v_{1},\ldots ,v_{m}\right)}^{⊤}|\tau^{2}\;\overset{i.i.d.}{∼}\;N\left(0,\tau^{2}\right) \end{array}$$ (2)

ICAR prior:


$$\begin{array}{l} v_{i}\;|\;{\left\{v_{j}\right\}}_{j\neq \;i},\;\tau^{2}∼\;N\left(\overset{m}{\underset{j=1}{\sum }}\frac{e_{ij}v_{j}}{e_{i+}\;},\frac{\tau^{2}}{e_{i+}}\right) \end{array}$$ (3)

where the number of neighbours that the ith vein segment has is denoted by $e_{i+}=\overset{m}{\underset{j=1}{\sum }}e_{ij}$. The ICAR prior (Eqn. 3) assumes smoothness between neighbouring vein segments’ frailty, that is, embolism formation of neighbouring spatial locations should occur around a similar time. In contrast, the IID prior (Eqn. 2) assumes the embolism events occur independently for different locations, disregarding any spatial correlations. This can lead to less accurate predictions when spatial dependencies are significant. However, the ICAR prior might overfit the data if the actual spatial dependency is weaker than anticipated, potentially leading to inaccurate results. To address this, we are adopting a data-driven approach to evaluate which model better fits the data, thereby gaining insights into how spatial proximity influences embolism propagation.

The final proposed model, named the ‘Spatial-Dependent-Vein-Thickness’ model, is a spatially dependent model that incorporates vein thickness. While the prior on the frailties is still the ICAR prior (Eqn. 3), the data and survival function are extended to include the vein thickness information (Eqn. 4). That is, the data for the Spatial-Dependent-Vein-Thickness model includes not only the adjacency matrix for the venation network (E), event indicator (δ), event water potential ($\tilde{t}$), but also vein thickness ($x=\left\{x_{i}:i\in 1,...,m\right\})$, where $x_{i}$ is the ith vein segment’s vein thickness. The Spatial-Dependent-Vein-Thickness model is built upon three main components: a basic temporal component $S_{0,\theta }\left(t\right)$, a spatial component $v_{i}$ (along with the ICAR prior), and a vein thickness component $\beta x_{i}$, with the corresponding survival function:


$$\begin{array}{l} S_{i}\;\left(t\right)=\frac{e^{-\left(v_{i}+\beta x_{i}\right)}\;S_{0,\theta }\left(t\right)}{1+\left(e^{-\left(v_{i}+\beta x_{i}\right)}-1\right)S_{0,\theta }\left(t\right)} \end{array}$$ (4)

The main parameters we’re estimating are the Weibull parameter (θ), which controls the temporal component; the spatial dependence parameter ($\tau^{-2}$), and the vein thickness regression coefficient (β). The Weibull base survival function $S_{\theta }\left(t\right)$ in the basic temporal component ($S_{0,\theta }\left(t\right))$ leverages the temporal information and governs the basic embolism progress over time for an entire leaf. Then, at the finer scale of vein segments, the spatial dependence parameter ($\tau^{-2}$) from the spatial component (Eqn. 3) accounts for the spatial smoothness of neighbouring vein segments. Greater values of the spatial dependence parameter indicate a higher likelihood of neighbouring vein segments undergoing embolism in a synchronized manner. Network connectivity is incorporated through the ICAR prior, capturing the spatial relationships within the vein network. Also, at the same scale, the vein thickness regression coefficient (β) from the vein thickness component determines the magnitude of the effect of the vein segment’s thickness on embolism dynamics. If the vein thickness regression coefficient is larger, it means vein thickness is a more crucial factor for predicting embolism propagation than spatial proximity.

### Model evaluation

We evaluated the performance of our proposed spatio-temporal models relative to the simple temporal model (KM estimator) by quantifying the amount of uncertainty reduced. The KM estimator (Kaplan and Meier 1958), a standard method in survival research, assumes no spatial dependence in embolism formation. Specifically, we calculated the 95% credible band area of the survival function for each model and quantified the relative change to the non-spatial model (KM estimator). If the spatial survival models successfully reduce the uncertainty of the estimates, the relative change should be more negative, meaning the spatial survival models produce a narrower credible band area on the survival probability estimates than the KM estimator.

We also evaluated the overall improvement of the two Spatial-Dependent-related models (i.e. Spatial-Dependent and Spatial-Dependent-Vein-Thickness models) over the Spatial-Independent model through a common model comparison criterion, the deviance information criterion (DIC) (Spiegelhalter et al. 2002). This criterion quantifies how well the model fits the data while penalizing model complexity.

We further compared how the two Spatial-Dependent-related models’ discrimination ability improved over the Spatial-Independent model through a concordance index (Harrell et al. 1996; Pencina and D’Agostino 2004). A comparable pair is a pair of vein segments with different event times. The concordance index (C-index) is defined as the probability that a random comparable pair of vein segments’ frailties (measured by log odds ratio), $v_{i}$ and $v_{j}$ is correctly ordered over the temporal evolution of embolism formation.


$$\begin{array}{l} P(v_{i}>v_{j}|t_{i}<t_{j}) \end{array}$$ (5)

The larger value of frailty implies an increased risk of embolizing. If the ith vein segment embolized earlier than the jth vein segment ($t_{i}<t_{j}$) and the model’s prediction is along the same direction as the observed event times, then the corresponding the ith vein segment’s frailty should be larger ($v_{i}>v_{j}$). We used the first 80% and the later 20% of observed embolism events as training and testing sets, respectively. The 80%–20% split, originating from the Pareto Principle, has been a well-supported practice for many empirical studies (Gholamy et al. 2018). To prevent overfitting, we calculated the C-index only on the testing set. During this particular evaluation set-up, models were trained on the training set only. We then estimated the C-index as the proportion of correctly ordered pairs among comparable pairs (i.e. ith vein segment is uncensored, $\delta_{i}=1$ and embolized earlier than the jth vein segment, $t_{i}<t_{j}$) from $m^{\prime }$ vein segments in the testing set:


$$\begin{array}{l} C=\frac{\overset{m^{\prime }}{\underset{i=1}{\sum }}\overset{m^{\prime }}{\underset{j=1}{\sum }}1_{v_{i}>v_{j}}\cdot 1_{t_{i}<t_{j}}\cdot \delta_{i}}{\overset{m^{\prime }}{\underset{i=1}{\sum }}\overset{m^{\prime }}{\underset{j=1}{\sum }}1_{t_{i}<t_{j}}\cdot \delta_{i}} \end{array}$$ (6)

This statistic summarizes the model’s ability to separate different responses for predictions along a time horizon. A higher C-index value means better model performance in predicting the order of embolism events. If the value is not significantly different from 0.5, then the model’s predictive ability is no better than random guessing. We compared performance between three spatio-temporal models (Spatial-Independent, Spatial-Dependent, and Spatial-Dependent-Vein-Thickness models) using two-way mixed analysis of variance (ANOVA) to determine if there are significant differences among at least two models. Once the difference was confirmed to be significant, we used paired *t*-tests with Bonferroni adjustments to understand which model had the best discrimination ability. More specifically, we compared the discrimination ability for a pair of two different models on the same sample to investigate which model has a better mean C-index, then we repeated the process on a different pair of models.

We assessed the relationship between vein features and the Spatial-Dependent-Vein-Thickness model parameters ($\beta ,\tau^{-2},\theta_{1},\theta_{2}$) through between-species Pearson correlation coefficients. We considered three vein features, including average vein density, the number of areoles per area, and degree of connectivity. The number of areoles per area provides a measure of the loopiness of the venation network (Blonder et al. 2011). The degree of connectivity is a common metric for summarizing how connected a transportation network is (Kansky 1963). In our study, we measure the connectivity of the venation network of leaf *l* as:


$$\begin{array}{l} Connectivity_{l}=\frac{No.\;branching\;points_{l}}{No.\;vein\;segments_{l}} \end{array}$$ (7)

The average number of branching points, average number of vein segments, average connectivity, and standard deviation of connectivity for each species are shown in Table 3.

**Table 3.** Summary statistics on vein thickness, number of branching points, number of vein segments, and degree of connectivity for each species. Each species code represents the following: ALCLAT, *Alchornea latifolia*; CASARB, *Casearia arborea*; CECSCH, *Cecropia schreberiana*; DRYGLA, *Drypetes glauca*; INGLAU, *Inga laurina*; OCOLEU, *Ocotea leucoxylon*; SLOBER, *Sloanea berteroana*; TABHET, *Tabebuia heterophylla*.

Code	Mean vein thickness	Average standard deviation vein thickness	Mean no. branching points	Mean no. vein segments	Mean connectivity	Standard deviation connectivity
ALCLAT	20.68	7.04	1088.75	1748.25	0.62	0.014
CASARB	19.31	7.22	392.13	686.50	0.56	0.033
CECSCH	18.18	9.31	971.57	1568.86	0.61	0.025
DRYGLA	26.69	13.7	122.43	244.14	0.50	0.021
INGLAU	24.22	6.37	375.17	649.50	0.57	0.014
OCOLEU	17.76	6.56	182.00	350.00	0.51	0.016
SLOBER	16.83	5.03	822.63	1372.50	0.60	0.022
TABHET	19.02	6.84	948.63	1539.75	0.61	0.027

We evaluated the relationship between hydraulic vulnerability, namely through the mean $P_{50}$ (MPa) obtained using the optical vulnerability technique (Smith-Martin et al. 2022, 2023) and different components of embolism propagation. Specifically, we calculated Pearson correlation coefficient between species P_**50**_ and the mean Spatial-Dependent-Vein-Thickness model parameters, including the mean vein thickness regression coefficient ($\bar{\beta }$), the mean spatial dependence parameter ($\bar{\tau^{-2}}$), and the mean Weibull base survival function parameters (${\bar{\theta }}_{1},{\bar{\theta }}_{2}$). Since our data is clustered into species, we decomposed the total correlation (i.e. the classic Pearson correlation coefficients computed at individual sample level) into between-species correlation and within-species correlation (Marzban *et al.* 2013). The total correlation is computed by neglecting the species structure of the data, assuming all samples are independent. However, this is not valid in our situation, where samples within the same species are dependent. Therefore, we considered either between-species correlation or within-species correlation, instead of total correlation. As our motivation is to see if we can predict a species’ mean parameter value or mean $P_{50}$, instead of whether a parameter is correlated with $P_{50}$ for a particular species, we focussed on between-species correlation. We compute between-species correlation using average parameter values and mean $P_{50}$ for each species.

## Results

### Visual comparison

All three models (the Spatial-Independent, Spatial-Dependent, and Spatial-Dependent-Vein-Thickness) estimated similar frailties for uncensored veins, with the primary, thicker veins embolizing first, followed by higher-order veins (Fig. 1e–g). As for the frailties of the censored vein segments, the Spatial-Dependent and Spatial-Dependent-Vein-Thickness models yielded similar estimates, whereas the Spatial-Dependent and Spatial-Independent models’ estimates were substantially different. As expected, because the ICAR prior imposes a smoothness assumption on vein segments that are connected, frailty values along a vein tend to be more similar compared with those using the IID prior. Nevertheless, the frailties of the Spatial-Independent model also picked up the broad venation structure on the uncensored veins, even without the smoothness constraint. This result reveals how venation structure naturally plays an important role in embolism formation and demonstrates why the smoothness introduced by the ICAR prior would be reasonable for modelling the spatial dependence of embolism formation.

### Do potential embolism propagation factors, such as vein thickness and spatial dependency, influence the estimation of xylem vulnerability curves and prediction of future embolism occurrences?

**Figure 3.**
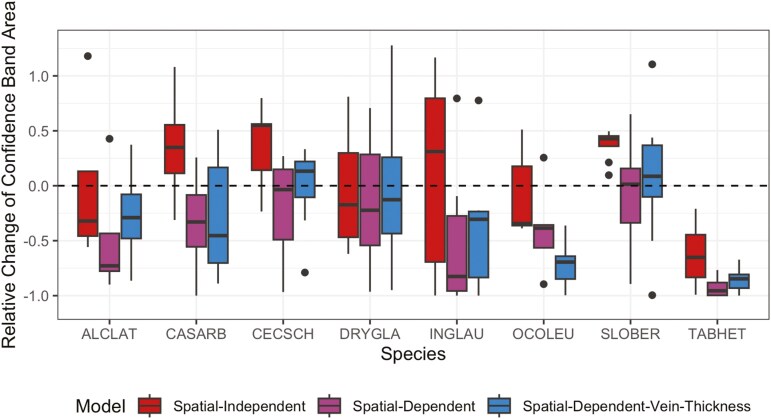
Relative change in credible band area with respect to the KM estimator for 3 models (the Spatial-Independent, Spatial-Dependent, and Spatial-Dependent-Vein-Thickness models) across species. If the relative change of credible band area is smaller, it means the model is more successful in reducing the uncertainty of estimating the survival curve. The models incorporating spatial smoothness (Spatial-Dependent and Spatial-Dependent-Vein-Thickness models) reduce uncertainty, suggesting that embolism propagation is influenced by spatial dependencies within the leaf venation network.

**Figure 4.**
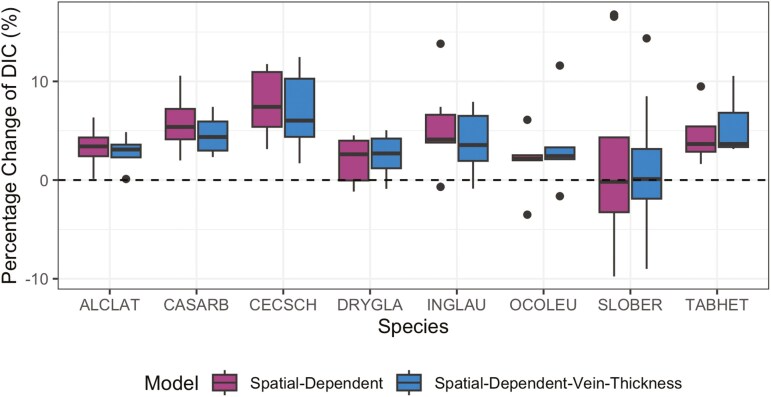
Relative improvement of the Spatial-Dependent-related models to the Spatial-Independent model for deviance information criterion (DIC) across species. Models that include spatial proximity (Spatial-Dependent and Spatial-Dependent-Vein-Thickness models) show improved model fit (DIC), indicating that accounting for spatial relationships enhances our understanding of how embolism dynamics propagate through the leaf network.

The models with the spatial smoothness constraint (the Spatial-Dependent and Spatial-Dependent-Vein-Thickness models) performed the best in reducing the uncertainty of the survival function’s credible band area estimation, implying embolism formation was spatially dependent. Depending on the species, the mean relative changes for the Spatial-Independent model were sometimes positive and sometimes negative, suggesting that this model does not reduce uncertainty compared to the non-spatial KM estimator (Fig. 3). In contrast, mean relative changes were mostly negative for all species using the two Spatial-Dependent-related models (except for *Cecropia schreberiana* and *Sloanea berteroana*), demonstrating that the spatial smoothness constraint reduces uncertainty in patterns of embolism formation. In addition, model comparison using the DIC supports the notion that the two Spatial-Dependent-related models are better than the Spatial-Independent model at capturing embolism formation patterns (Fig. 4), with the exception for *Sloanea berteroana*.

Vein thickness information improved the prediction of future embolism events when the spatial dependency of embolism formation is considered. The superior performance of the Spatial-Dependent-Vein-Thickness model in predicting the correct order of embolism events was supported by the data. The Spatial-Dependent-Vein-Thickness model had the highest mean C-index (*M* = 0.542, SD = 0.151) and had significantly higher mean C-index than the other two models. The interaction term between model types and species in explaining the C-index was not statistically significant (*F* (8.09, 48.54) = 1.65, *P* = .13), ascertaining that the effect of model type on C-index is similar for different species. There was a statistically significant difference in C-index (*F* (1.16, 48.54) = 9.314, *P* = .003) between at least two models among the three spatial survival models (the Spatial-Independent, Spatial-Dependent, and Spatial-Dependent-Vein-Thickness models). Pairwise comparisons revealed that the mean C-index was significantly different in Spatial-Dependent-Vein-Thickness vs. Spatial-Dependent comparison (*t* (49) = −3.78, *P* = .001); in Spatial-Dependent-Vein-Thickness vs. Spatial-Independent (t (49) = −3.66, *P* = .002), but not in Spatial-Dependent vs. Spatial-Independent (*t* (49) = −2.15, *P* = .108).

### What are the relationships between the spatio-temporal dynamics of embolism propagation and leaf venation features?

**Figure 5.**
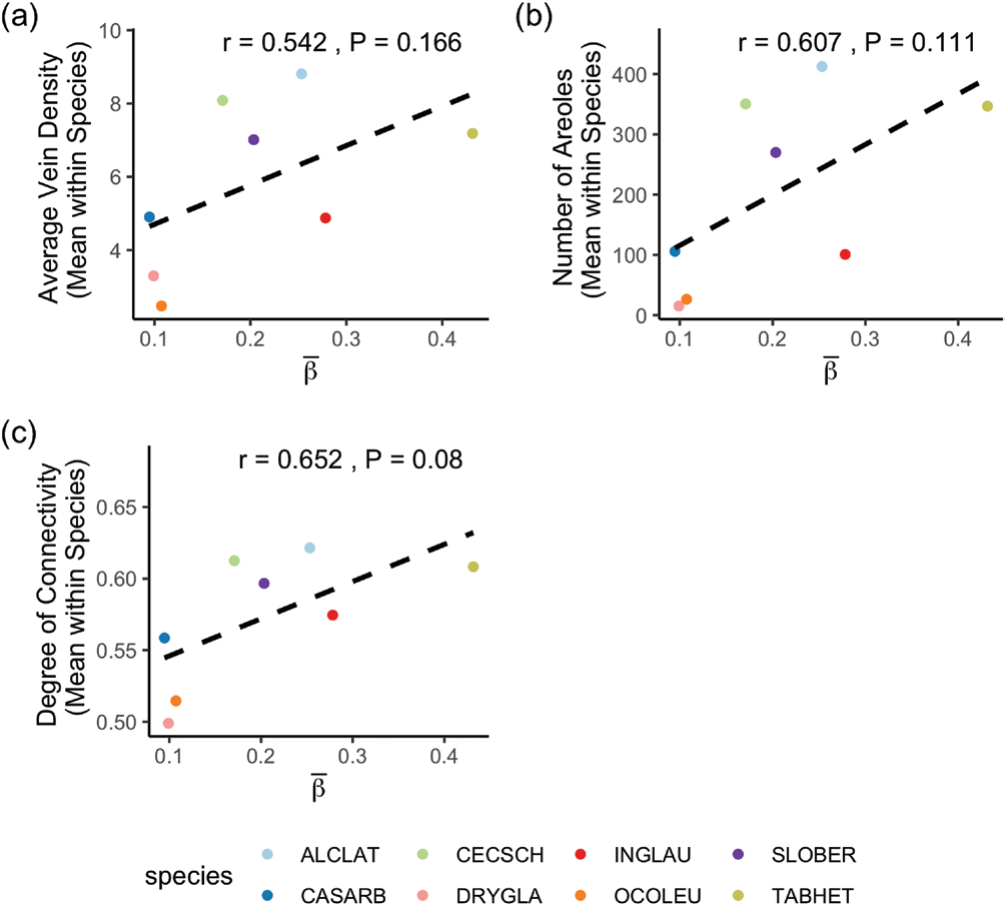
Relationship between vein thickness regression coefficient from the Spatial-Dependent-Vein-Thickness model and (a) Average vein density, (b) Number of areoles per area, and (c) Degree of connectivity. The dashed line shows the linear regression results. Each species code represents the following: ALCLAT, *Alchornea latifolia*; CASARB, *Casearia arborea*; CECSCH, *Cecropia schreberiana*; DRYGLA, *Drypetes glauca*; INGLAU, *Inga laurina*; OCOLEU, *Ocotea leucoxylon*; SLOBER, *Sloanea berteroana*; TABHET, *Tabebuia heterophylla*.

Our analysis, based on the Spatial-Dependent-Vein-Thickness model, revealed a significant positive correlation between the vein thickness regression coefficient ($\bar{\beta }$) and the degree of vein connectivity (Fig. 5), while the other parameters ($\bar{\tau^{-2}},\bar{\theta_{1}},\bar{\theta_{2}}$) were not significantly correlated with any of the three vein features (average vein density, number of areoles, and degree of vein connectivity) considered. In other words, if veins are more connected with one another, $\bar{\beta }$ would be larger, making the importance of vein thickness in embolism progression stronger. This finding indicates that vein connectivity plays a crucial role in amplifying the influence of vein thickness on embolism propagation, highlighting a previously unexplored relationship between leaf venation features and the spatio-temporal dynamics of embolism.

**Figure 6.**
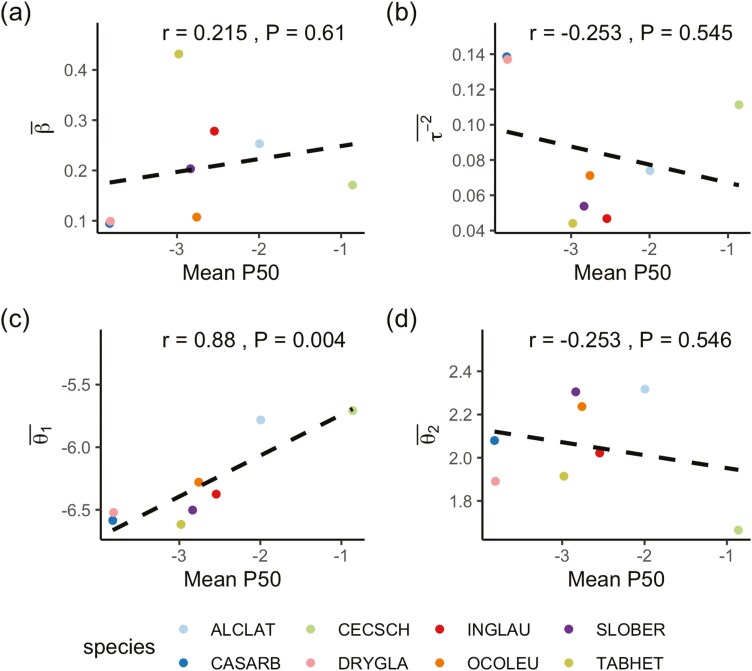
Relationship between mean $P_{50}$ (MPa) from Smith-Martin *et al.* 2022, 2023 and mean parameters from the Spatial-Dependent-Vein-Thickness model: (a) Vein thickness regression coefficient. (b) Spatial dependence parameter. (c) and (d) The Weibull parameters for the Weibull base survival function $S_{\theta }\left(t\right)$, $\theta_{1}$ controls the scale and $\theta_{2}$ oversees the shape. Positive values of $\theta_{2}$ indicate that vein segments are more likely to embolize as time progresses. The more negative $\theta_{1}$ is, the more variability will be presented for the Weibull distribution. Each species code represents the following: ALCLAT, *Alchornea latifolia*; CASARB, *Casearia arborea*; CECSCH, *Cecropia schreberiana*; DRYGLA, *Drypetes glauca*; INGLAU, *Inga laurina*; OCOLEU, *Ocotea leucoxylon*; SLOBER, *Sloanea berteroana*; TABHET, *Tabebuia heterophylla*.

We also found that  is relatively large for certain species (Fig. 6a), such as *Tabebuia heterophylla*, indicating vein thickness is a critical factor for embolism propagation in this species, rather than spatial proximity. In contrast,  is relatively small (close to 0) for other species such as *Drypetes glauca* and *Casearia arborea*, implying vein thickness has little impact on embolism propagation. In future work, analysis of microscopic anatomical structure with scanning electron microscopy (SEM)/transmission electron microscopy (TEM) could provide additional insights about embolism propagation across species.

### Can our spatial survival model validate findings from existing literature?

**Figure 7.**
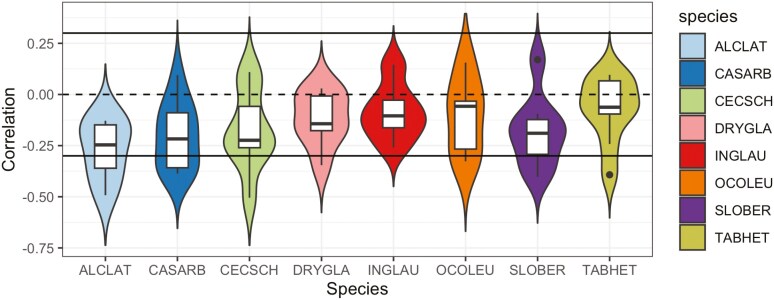
Pearson correlation coefficient between vein thickness and water potential of embolism events for different species. Correlations were estimated separately for each leaf sample. Correlation values between 0.3 and −0.3 are considered weak correlations. The more negative the correlation is, the stronger the pattern of embolisms propagating from thicker veins to thinner veins. Each species code represents the following: ALCLAT, *Alchornea latifolia*; CASARB, *Casearia arborea*; CECSCH, *Cecropia schreberiana*; DRYGLA, *Drypetes glauca*; INGLAU, *Inga laurina*; OCOLEU, *Ocotea leucoxylon*; SLOBER, *Sloanea berteroana*; TABHET, *Tabebuia heterophylla*.

The average vein thickness and average standard deviation of vein thickness for each species are summarized (Table 3). A total of 42 out of 53 samples had a negative correlation between vein thickness and water potential at which embolism formation occurs, suggesting that a smaller vein size lowers the risk of embolism formation (Fig. 7). Although the weak correlations (|*r*|<0.3) for most samples implied that vein size alone cannot explain patterns of embolism formation, results suggest that integrating vein thickness information into models is important.

The posterior mean of the vein thickness regression coefficient (β) for each leaf sample from the Spatial-Dependent-Vein-Thickness model was significantly positive for most cases (47 samples out of a total of 53 samples), suggesting that thicker veins embolize earlier. Our finding is purely data-driven because we did not impose any prior knowledge that assumes the regression coefficient is more likely to be positive. Nonetheless, it aligns with observations reported in the existing literature.

Our analysis revealed a significant between-species correlation between hydraulic vulnerability (mean *P*_50_) and the Weibull scale parameter ($\theta_{1}$) (Fig. 6c), while there were no significant between-species correlations with other parameters derived from our analyses (Fig. 6a, b, d). These findings suggest that the basic temporal component's Weibull base survival function $S_{\theta }\left(t\right)$, which encodes information similar to the mean $P_{50}$, is primarily concerned with embolism propagation over time. On the other hand, the spatial component and vein thickness component utilize additional information to describe the spatial and vein thickness aspects of embolism propagation.

## Discussion

Our proposed spatial survival model captures embolism propagation at an exceptionally fine scale (i.e. leaf vein segments). This fine-grained approach allowed us to explore the spatial dependence between adjacent veins in the context of embolism formation, allowing us to investigate into the complex relationship between spatial proximity, vein thickness, and network connectivity. Furthermore, our model explicitly disentangles the role of distinct driving factors, namely temporal, spatial dependence, and vein thickness, on embolism propagation, offering the ability to gauge the individual contributions of these factors and uncover new insights between these factors and leaf venation features.

Our results reveal that spatial dependency reduces the posterior predictive error (DIC) and uncertainty in survival estimation, implying that embolism probability increases when neighbouring veins embolize. Moreover, incorporating vein thickness information improved the prediction of future embolism events when the spatial dependency was considered. We also found that species with high vein network connectivity exhibited a more pronounced embolization pattern from thicker to thinner veins. Although the underlying biological mechanism remains unclear, we anticipate that this relationship will be subject to further investigation.

Our findings align with previous analyses of embolism formation that do not incorporate spatial data. Specifically, we found that the main, thicker veins embolized first, followed by higher-order veins, confirming similar results for tropical evergreen tree species (Brodribb *et al.* 2016a). Regarding the debate about the influence of vein thickness on embolism (Anfodillo and Olson 2021; Lens et al. 2022), our results support the view that vein thickness is positively associated with $P_{50}$ (Maherali et al. 2006; Fu et al. 2012; Hacke et al. 2017). Additionally, our temporal component exhibited a significant correlation with the mean *P*_50_, signifying that it encapsulates meaningful temporal insights from the data.

Our model serves as a pioneering step in spatial-dependent embolism propagation modelling at a finer scale, with the potential for encompassing additional factors. By evaluating the corresponding coefficients, one can discern the significance of these additional factors on embolism propagation. With the adoption of more sophisticated modelling techniques, we can attain a deeper understanding of the dynamics underpinning embolism formation.

### Survival analysis

As embolism formation is in alignment with the assumption of survival analysis, we fitted three survival models in our analysis. Each of them leveraged different information (temporal, spatial, or vein thickness information), or assumptions on the spatial dependency of embolism formation. Comparative analysis of model performances confirmed the presence of spatial dependency in embolism formation, revealing how different factors contribute to the improvement in estimation and prediction. To the best of our knowledge, this is the first study that has utilized survival models to address this issue.

One limitation of our study is our analysis of vein segments from images rather than xylem conduits, and it is possible that one xylem conduit is being separated into multiple vein segments or that one vein segment contains multiple conduits (Mrad et al. 2021; Bouda et al. 2022). This approximation on the analysis unit might bias the spatial dependence parameter. However, this is the best approximation we could get at the current resolution of images obtained through the optical vulnerability technique. Our survival models can be applied to finer-resolution images as they become available, allowing for more accurate modelling of the xylem network and improved estimates of spatial dependence by shifting from vein segments to xylem conduits.

Another limitation is the choice of priors used in our model, as the true underlying spatial dependency mechanism in embolism formation is not fully known. While ICAR priors outperformed IID priors on our dataset, they may oversimplify or overly complicate the spatial interactions involved, suggesting that exploring alternative priors could be a promising direction for future research.

### Pre-existing embolisms increase the likelihood of embolism formation in neighbouring veins

We found that pre-existing embolisms increase the probability of embolism formation in neighbouring leaf veins. Our findings suggest that embolisms are spreading from embolized vessels into adjacent water-filled vessels. Brodribb et al. (2016a) discussed this through visual cues, whereas our study provides model-based evidence that can be scaled to large datasets. This highlights the importance of spatial dependency in embolism propagation, and our approach lays the foundation for future studies to explore how spatial dependence varies across species and influences embolism dynamics.

### Leaf venation network features and embolism propagation

Our findings support the importance of vein thickness in embolism progression, consistent with existing literature (Brodribb *et al.* 2016a; Skelton *et al.* 2021). Thicker veins embolized earlier in our study, reflected by the negative correlation with water potential and by the positive vein thickness regression coefficients for most individuals. This supports existing literature (Maherali et al. 2006; Fu et al. 2012; Hacke et al. 2017; Isasa et al. 2023) within the context of ongoing debate (Anfodillo and Olson 2021; Lens et al. 2022).

When the spatial dependency of embolism formation was considered, incorporating vein thickness information improved the prediction of future embolism events. Moreover, the vein thickness regression coefficient quantified its influence on embolism progression compared to spatial proximity. We observed that the impact of vein thickness varies across species, suggesting future work could investigate the microscopic anatomical structure using SEM/TEM to gain additional insights.

We found that the vein thickness regression coefficient was positively correlated with vein network connectivity, implying that the pattern of embolizing from thicker to thinner veins is stronger in highly connected networks. This finding could help predict species’ vulnerability to embolism based on leaf network connectivity, which can be readily obtained from field data or museum specimens. By leveraging automated tools for extracting venation metrics (e.g. vein looping, centrality), our approach can be extended to investigate their influence on embolism propagation in large datasets.

Our approach introduces a novel method for studying embolism propagation by isolating key driving factors and modelling the influence of vein thickness. This allows us to explore the interactions between vein thickness, spatial proximity, and leaf venation features—an aspect largely overlooked in previous literature (Brodribb *et al.* 2016a; Hacke et al. 2017; Johnson et al. 2018; Isasa et al. 2023).

## Supplementary Material

plaf020_suppl_Supplementary_Materials

## Data Availability

The mean P50 of the optical vulnerability curves is available from https://doi.org/10.1111/nph.18175 (Smith-Martin et al. 2022). The image data, processed data, and code for the survival models are available at https://doi.org/10.5281/zenodo.15049079.

## References

[CIT0001] Anfodillo T, Olson ME. Tree mortality: testing the link between drought, embolism vulnerability, and xylem conduit diameter remains a priority. Front For Glob Change 2021;4:704670. 10.3389/ffgc.2021.704670

[CIT0002] Blonder B, Violle C, Bentley LP et al Venation networks and the origin of the leaf economics spectrum. Ecol Lett 2011;14:91–100. 10.1111/j.1461-0248.2010.01554.x 21073643

[CIT0003] Blonder B, Both S, Jodra M et al Linking functional traits to multiscale statistics of leaf venation networks. New Phytol 2020;228:1796–810. 10.1111/nph.16830 32712991

[CIT0004] Bouda M, Huggett BA, Prats KA et al Hydraulic failure as a primary driver of xylem network evolution in early vascular plants. Science 2022;378:642–6. 10.1126/science.add2910 36356120

[CIT0005] Boyer JS. Water transport. Annu Rev Plant Physiol 1985;36:473–516. 10.1146/annurev.pp.36.060185.002353

[CIT0007] Brodribb TJ, Bienaimé D, Marmottant P. Revealing catastrophic failure of leaf networks under stress. Proc Natl Acad Sci USA 2016a;113:4865–9. 10.1073/pnas.1522569113 27071104 PMC4855591

[CIT0008] Brodribb TJ, Skelton RP, McAdam SAM et al Visual quantification of embolism reveals leaf vulnerability to hydraulic failure. New Phytol 2016b;209:1403–9. 10.1111/nph.13846 26742653

[CIT0009] Bühler J, Rishmawi L, Pflugfelder D et al phenoVein—a tool for leaf vein segmentation and analysis. Plant Physiol 2015;169:2359–70. 10.1104/pp.15.00974 26468519 PMC4677892

[CIT0012] Dixon, H.H. (1914) Transpiration and the Ascent of Sap in Plants. London: Macmillan Co. Ltd.

[CIT0013] Dixon HH, Joly J. On the ascent of sap. Philos Trans R Soc Lond. B 1895;186:563–76.

[CIT0014] Ewel, J.J. & Whitmore, J.L. (1973) The Ecological Life Zones of Puerto Rico and the U.S. Virgin Islands. Rio Piedras, P.R: Institute of Tropical Forestry.

[CIT0015] Fu P-L, Jiang Y-J, Wang A-Y et al Stem hydraulic traits and leaf water-stress tolerance are coordinated with the leaf phenology of angiosperm trees in an Asian tropical dry karst forest. Ann Bot (Lond) 2012;110:189–99. 10.1093/aob/mcs092 PMC3380589 22585930

[CIT0050] Gholamy A, Kreinovich V, Kosheleva O. Why 70/30 or 80/20 relation between training and testing sets: a pedagogical explanation. Int J Intel Technol Appl Stat 2018;11:105–11. 10.6148/IJITAS.201806_11(2).0003

[CIT0017] Hacke UG, Sperry JS, Wheeler JK et al Scaling of angiosperm xylem structure with safety and efficiency. Tree Physiol 2006;26:689–701. 10.1093/treephys/26.6.689 16510385

[CIT0018] Hacke UG, Spicer R, Schreiber SG et al An ecophysiological and developmental perspective on variation in vessel diameter. Plant Cell Environ 2017;40:831–45. 10.1111/pce.12777 27304704

[CIT0019] Harrell FE Jr., Lee KL, Mark DB. Multivariable prognostic models: issues in developing models, evaluating assumptions and adequacy, and measuring and reducing errors. Stat Med 1996;15:361–87. 10.1002/(SICI)1097-0258(19960229)15:4<361::AID-SIM168>3.0.CO;2-4 8668867

[CIT0020] Hougaard P. Frailty models for survival data. Lifetime Data Anal 1995;1:255–73. 10.1007/BF00985760 9385105

[CIT0021] Isasa E, Link RM, Jansen S et al Addressing controversies in the xylem embolism resistance–vessel diameter relationship. New Phytol 2023;238:283–96. 10.1111/nph.18731 36636783

[CIT0022] Johnson KM, Jordan GJ, Brodribb TJ. Wheat leaves embolized by water stress do not recover function upon rewatering. Plant Cell Environ 2018;41:2704–14. 10.1111/pce.13397 29981153

[CIT0023] Kansky, K.J. (1963) The Structure of Transport Networks. Chicago, USA: University of Chicago.

[CIT0024] Kaplan EL, Meier P. Nonparametric estimation from incomplete observations. J Am Stat Assoc 1958;53:457–81. 10.2307/2281868

[CIT0025] Lens F, Gleason SM, Bortolami G et al Functional xylem characteristics associated with drought-induced embolism in angiosperms. New Phytol 2022;236:2019–36. 10.1111/nph.18447 36039697

[CIT0026] Leung K-M, Elashoff RM, Afifi AA. Censoring issues in survival analysis. Annu Rev Public Health 1997;18:83–104. 10.1146/annurev.publhealth.18.1.83 9143713

[CIT0027] Maherali H, Moura CF, Caldeira MC et al Functional coordination between leaf gas exchange and vulnerability to xylem cavitation in temperate forest trees. Plant Cell Environ 2006;29:571–83. 10.1111/j.1365-3040.2005.01433.x 17080608

[CIT0049] Marzban C, Illian PR, Morison D Mourad PD. Within-group and between-group correlation: illustration on non-invasive estimation of intracranial pressure. 2013. viewed nd, from http://faculty.washington.edu/marzban/within_between_simple.pdf

[CIT0030] Mrad A, Johnson DM, Love DM et al The roles of conduit redundancy and connectivity in xylem hydraulic functions. New Phytol 2021;231:996–1007. 10.1111/nph.17429 33908055

[CIT0031] Pencina MJ, D’Agostino RB. Overall C as a measure of discrimination in survival analysis: model specific population value and confidence interval estimation. Stat Med 2004;23:2109–23. 10.1002/sim.1802 15211606

[CIT0033] Sack L, Holbrook NM. Leaf hydraulics. Annu Rev Plant Biol 2006;57:361–81. 10.1146/annurev.arplant.56.032604.144141 16669766

[CIT0034] Sack L, Scoffoni C. Leaf venation: structure, function, development, evolution, ecology and applications in the past, present and future. New Phytol 2013;198:983–1000. 10.1111/nph.12253 23600478

[CIT0035] Scholander PF. Tensile water: cohesiveness of water under negative pressure is a key to understanding the mechanism of imbibition and osmosis. Am Sci 1972;60:584–90.

[CIT0036] Scoffoni C, Albuquerque C, Brodersen CR et al Leaf vein xylem conduit diameter influences susceptibility to embolism and hydraulic decline. New Phytol 2017;213:1076–92. 10.1111/nph.14256 27861926

[CIT0038] Skelton RP, Brodribb TJ, Choat B. Casting light on xylem vulnerability in an herbaceous species reveals a lack of segmentation. New Phytol 2017;214:561–9. 10.1111/nph.14450 28124474

[CIT0037] Skelton RP, Anderegg LDL, Diaz J et al Evolutionary relationships between drought-related traits and climate shape large hydraulic safety margins in western North American oaks. Proc Natl Acad Sci USA 2021;118:e2008987118. 10.1073/pnas.2008987118 33649205 PMC7958251

[CIT0039] Smith-Martin CM, Muscarella R, Ankori‐Karlinsky R et al Hurricanes increase tropical forest vulnerability to drought. New Phytol 2022;235:1005–17. 10.1111/nph.18175 35608089

[CIT0040] Smith-Martin CM, Muscarella R, Ankori‐Karlinsky R et al Hydraulic traits are not robust predictors of tree species stem growth during a severe drought in a wet tropical forest. Funct Ecol 2023;37:447–60. 10.1111/1365-2435.14235

[CIT0041] Spiegelhalter DJ, Best NG, Carlin BP et al Bayesian measures of model complexity and fit. J R Stat Soc Ser B Stat Methodol 2002;64:583–639. 10.1111/1467-9868.00353

[CIT0042] Tyree MT, Ewers FW. The hydraulic architecture of trees and other woody plants. New Phytol 1991;119:345–60. 10.1111/j.1469-8137.1991.tb00035.x

[CIT0043] Tyree MT, Sperry JS. Vulnerability of xylem to cavitation and embolism. Annu Rev Plant Physiol Plant Mol Biol 1989;40:19–36. 10.1146/annurev.pp.40.060189.000315

[CIT0044] van der Walt S, Schönberger JL, Nunez-Iglesias J et al; scikit-image contributors. scikit-image: image processing in Python. PeerJ 2014;2:e453. 10.7717/peerj.453 25024921 PMC4081273

[CIT0045] Watt DC, Aitchison TC, MacKie RM et al Survival analysis: the importance of censored observations. Melanoma Res 1996;6:379–85. 10.1097/00008390-199610000-00005 8908598

[CIT0046] Xu H, Blonder B, Jodra M et al Automated and accurate segmentation of leaf venation networks via deep learning. New Phytol 2021;229:631–48. 10.1111/nph.16923 32964424

[CIT0047] Zhou H, Hanson T, Zhang J. spBayesSurv: fitting Bayesian spatial survival models using R. J Stat Softw 2020;92:1–33. 10.18637/jss.v092.i09

[CIT0048] Zimmermann, M.H. (1983) Xylem Structure and the Ascent of Sap. Springer: Berlin, Heidelberg Germany.

