# Supplementary material for: A spatio-temporal model of embolism propagation in leaf vein networks
Source: AoB Plants. 2025 Apr 12;17(4):plaf020. doi: 10.1093/aobpla/plaf020 (PMC12282127; doi:10.1093/aobpla/plaf020)
Supplement: plaf020_suppl_Supplementary_Materials [file plaf020_suppl_supplementary_materials.pdf]

SUPPORTING INFORMATION

Figure. S1: Close-up views of vein segmentation using phenoVein.

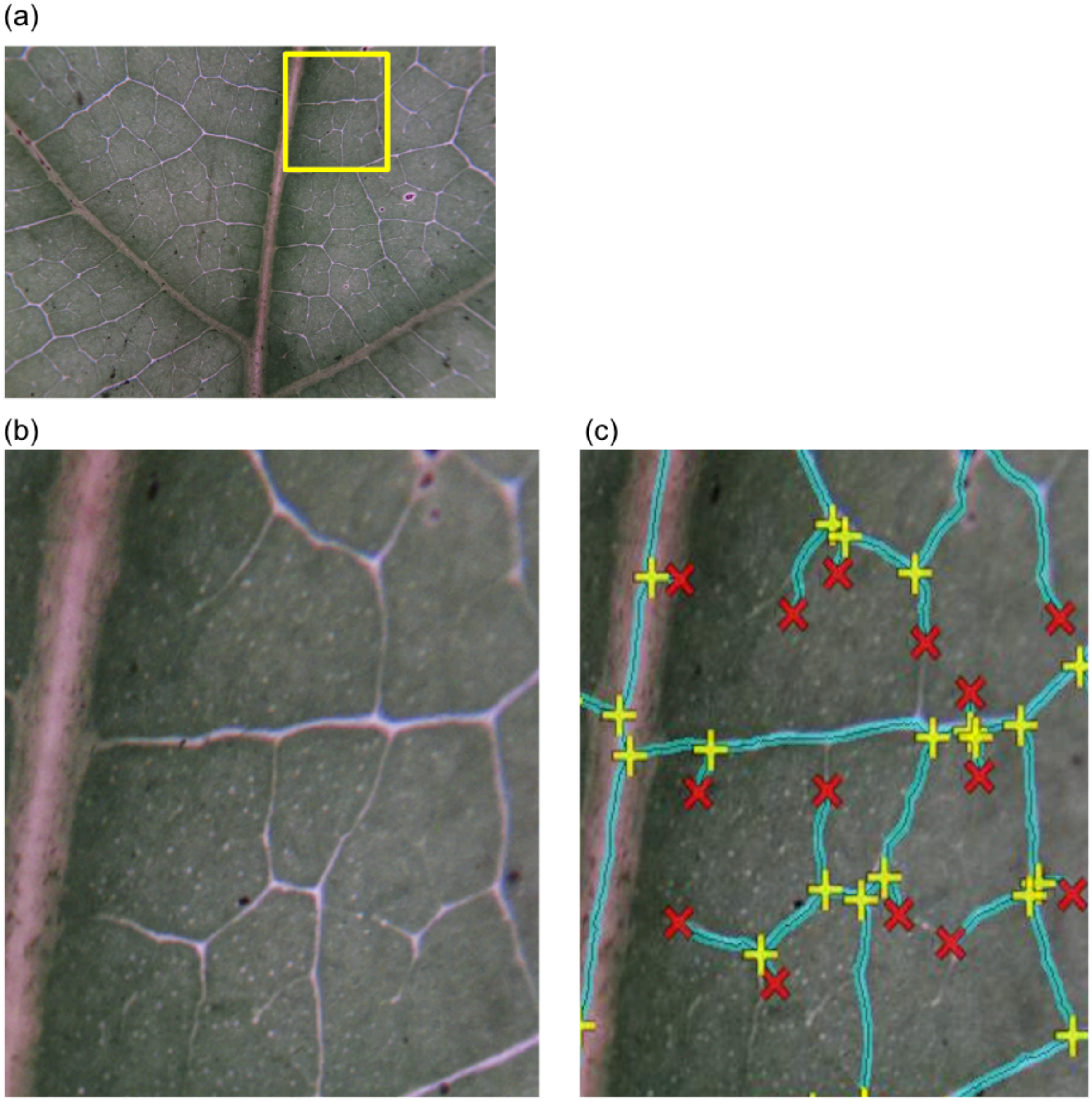

Fig. S1 Close-up views of vein segmentation using phenoVein. (a) Unprocessed image of an *Ocotea leucoxylon* sample, with the yellow box indicating the area that has been zoomed in. (b) Magnified view of the region highlighted by the yellow box in (a). (c) The corresponding vein segmentation marked by phenoVein.

Notes S1: Mapping Embolized Pixels to Embolism Events

When mapping embolized pixels to embolism events, several considerations are important. Since embolized pixels are extracted by human annotators using optical vulnerability techniques, the data is generally clean, with minimal obvious false positives. However, embolized pixels may span multiple vein segments, and there could be minor spillovers to adjacent vein segments. To address these issues, we use the following strategy for each image with embolized pixels:

1. **Identify Candidates**: All vein segments containing at least one embolized pixel are considered "candidates."
2. **Calculate Overlapping Percentage**: We define the "overlapping percentage" as the ratio of the intersection between embolized pixels and the pixels of a vein segment candidate to the total number of embolized pixels. This percentage represents how much of the embolized area is associated with each vein segment candidate.
3. **Iterative Removal**: We iteratively remove candidates with overlapping percentages below 5%, in ascending order of their percentages under the constraint that the remaining candidates must collectively account for at least 90% of the total overlapping percentage.
4. **Final Selection**: The remaining vein segment candidates, after applying this process, are classified as experiencing one embolism event.

Notes S2: Vulnerability Curves and Survival Functions

Our choice to use a survival model is driven by the nature of embolism occurrence. Once xylem vessel conduits are embolized, they lose their ability to transport water, analogous to how a patient ceases to survive once they have died. In this section, we further justify our use of a survival model by establishing a connection between survival functions and vulnerability curves.

Vulnerability curves describe the relationship between the percentage loss of xylem conductance (PLC) and xylem water potential (Ψ). Assuming the Weibull formulation of relative hydraulic conductance, the PLC can be expressed as:

$$PLC(\Psi) = 1 - e^{-\left(-\frac{\Psi}{\theta_1}\right)^{\theta_2}}$$

Here $\theta_1 > 0$ and $\theta_2 > 0$ represent the scale and shape parameters of a Weibull distribution. If the survival time also follows a Weibull distribution with the same parameters, the survival function is:

$$S_\theta(t) = e^{-\left(\frac{t}{\theta_1}\right)^{\theta_2}}$$

Essentially, this survival function mirrors the relative conductance in PLC when time in the survival function is replaced by the negative of water potential. Therefore, under the assumption that relative conductance and survival time follow the same distribution (*e.g.*, Weibull distribution), the vulnerability curve (VC) obtained using is equivalent to 1− the survival function.

This connection demonstrates that the spatial survival model we proposed aligns with established xylem analysis frameworks. By linking survival functions to vulnerability curves, we reinforce that our approach is grounded in well-understood principles of xylem hydraulics.
